# Activator of apoptosis harakiri (HRK) localisation at mitochondria alters mitochondrial morphology independently of other BCL‐2 proteins

**DOI:** 10.1111/febs.70255

**Published:** 2025-09-12

**Authors:** Louise E. King, Lukas Faber, Ana J. García‐Sáez

**Affiliations:** ^1^ Institute for Genetics, CECAD Research Center University of Cologne Cologne Germany; ^2^ Department of Membrane Dynamics Max Planck Institute of Biophysics Frankfurt Germany

**Keywords:** apoptosis, BH3, Harakiri, mitochondria, transmembrane

## Abstract

The activator of apoptosis harakiri (HRK) is a pro‐apoptotic BCL‐2 homology 3 (BH3)‐only protein of the apoptosis regulator Bcl‐2 (BCL‐2) family that is mainly expressed in neuronal and haematopoietic tissues. How specific HRK protein domains contribute to its pro‐apoptotic function, and what other non‐apoptotic roles HRK performs within cells, remain poorly understood. Here, we evaluated the apoptosis sensitivity, and mitochondrial shape and function of HCT116 human colorectal cells lacking all BH3‐only proteins as well as all relevant BCL‐2 proteins. By reconstituting individual BH3‐only proteins on this genetic background, we observed that HRK induces apoptosis in a manner dependent on its BH3 domain, and the presence of the apoptosis regulator BAX and BCL‐2 homologous antagonist/killer (BAK), but independent of its transmembrane domain. Intriguingly, HRK also causes mitochondrial aggregation without altering cristae structure or respiration. Although the BH3 domain is not required for mitochondrial reorganisation, we found that the transmembrane domain requires additional upstream amino acids for HRK mitochondrial localisation and reorganisation. These observations uncover a previously unknown role of HRK in modulating mitochondrial morphology that is independent of its BH3 domain and pro‐death function.

## Graphical abstract

How the structure of HRK, a member of the BCL‐2 protein family, contributes to its function is poorly understood. Functional analysis of HRK mutants reveals that HRK induces apoptosis in a BH3‐domain‐dependent, but transmembrane‐domain‐independent, manner. The transmembrane domain localises HRK to mitochondria, but alone is insufficient. These observations uncover a previously unknown role of HRK in modulating mitochondrial morphology that is independent of its BH3 domain and pro‐death function.

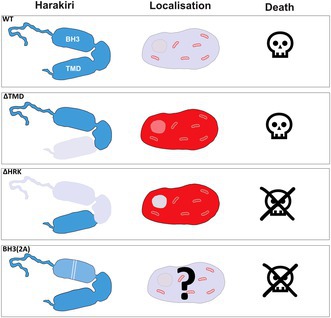

**Abbreviations**
BAD: BCL‐2 antagonist of cell death
BAK: BCL‐2 antagonist/killer 1
BAX: BCL‐2 associated X, apoptosis regulator
BCL: B cell lymphoma
BCL‐XL: BCL‐2‐like protein 1
BH: BCL‐2 homology
BID: BH3‐interacting domain death agonist
BIK: BCL‐2 interacting killer
BIM: BCL‐2 interacting mediator of cell death
BMF: BCL‐2 modifying factor
BOK: BCL‐2 related ovarian killer
C1QBP/p32: complement C1q binding protein
CLEM: correlative light emission microscopy
CTCF: corrected total cell fluorescence
DIVA: death inducer binding to VBCL‐2 and APAF‐1
DREAM: downstream regulatory element antagonist modulator
ECAR: extracellular acidification rate
EGFP: enhanced green fluorescent protein
FACS: fluorescence activated cell sorting
FL: full‐length
HRK: Harakiri
KO/DKO: knockout/double knockout
MCL‐1: induced myeloid leukaemia cell differentiation protein
MERCS: mitochondrial‐endoplasmic reticulum contact site
MIM: mitochondrial inner membrane
MOM: mitochondrial outer membrane
MOMP: mitochondrial outer membrane permeabilization
OCR: oxygen consumption rate
OXPHOS: oxidative phosphorylation
PUMA: P53‐upregulated modulator of apoptosis
STED: stimulated emission depletion
TEM: transmission electron tomography
TM: transmembrane
WT: wildtype

## Introduction

The BCL‐2 family of proteins regulates apoptosis via interaction between family members and the mitochondrial outer membrane (MOM). All members of the family share at least one homologous region within their structure termed a BCL‐2 homology (BH) domain and are divided into three categories based on their structure and function: anti‐apoptotic or guardians, pro‐apoptotic BH3‐only proteins or initiators, and executioners [1]. The anti‐apoptotic proteins contain four of these regions – BH1‐BH4 – as well as a C‐terminal transmembrane (TM) domain required for localisation at the MOM. Anti‐apoptotic proteins can bind to and inhibit the pro‐apoptotic proteins, maintaining cell viability [2]. The executioner proteins, like BAX and BAK, are very similar in structure to the guardian proteins, but function to induce apoptosis by oligomerising and forming pores in the MOM to induce MOM permeabilisation (MOMP) [3]. This releases factors such as cytochrome c from the mitochondrial intermembrane space, which forms the apoptosome with the protein apoptotic protease‐activating factor 1 (APAF‐1), triggering a downstream caspase signalling cascade and initiating the compartmentalisation and destruction of the cell. Finally, the pro‐apoptotic BH3‐only proteins, as their name suggests, contain only a single BH domain, the BH3 domain, and function to either sequester the anti‐apoptotic proteins (termed sensitisers) or also to activate the effectors (termed direct activators), generally increasing the overall pro‐apoptotic signal at the MOM [4].

Whilst our knowledge of the fundamental steps of apoptosis and how BCL‐2 proteins regulate it has been known for a number of years, the specific role each individual family member plays is still unclear [5, 6]. The BCL‐2 family is relatively large, with over 20 members [7] and as such, many of the proteins show a degree of functional redundancy. Whilst the binding specificity between different family members leads to differential apoptotic responses [8], it is perhaps unsurprising that alternative functions of BCL‐2 proteins have also been identified. Both anti‐apoptotic and effector BCL‐2 proteins are involved in processes including regulating mitochondrial morphology, the DNA damage response, lipid and glucose metabolism, calcium homeostasis, and the unfolded protein response [9]. BH3‐only proteins, which structurally speaking are much simpler than other BCL‐2 family members, also have roles in alternative, non‐apoptotic functions. For example, BCL‐2‐associated agonist of cell death (BAD) has a known role in glucose metabolism [10, 11], BH3‐interacting domain death agonist (BID) in lipid transport [12], and phorbol‐12‐myristate‐13‐acetate‐induced protein 1 (NOXA) in autophagy [13, 14]. One such BH3‐only protein, HRK, is less studied than the others, with comparatively few structural or functional studies to date.

HRK was discovered via a yeast two‐hybrid assay identifying novel proteins involved in apoptotic regulation, using BCL‐2 as bait [15]. Within the same study, HRK was shown to induce cell death via interaction with BCL‐2‐like protein 1 (BCL‐XL) but not BAX, BAK, or the shorter isoform of BCL‐XL, BCL‐XS (although later studies did confirm BAX is indeed required [16]). This has since been confirmed via fluorescence polarisation binding assay, showing a relatively high binding affinity between BCL‐XL and HRK BH3 binding peptide [17] as well as in systematic KO studies showing HRK promiscuously binds both BCL‐XL and induced myeloid leukaemia cell differentiation protein (MCL‐1) [18]. The HRK mouse homologue, DP5, was also shown to interact with BCL‐XL upon neuronal cell death [19]. These interactions with anti‐apoptotic proteins occur via the HRK BH3 domain, placing it in the sensitiser category of BH3‐only proteins. HRK is predominantly expressed in lymphoid tissues, as well as bone marrow, the pancreas and the spleen [15]. Much of the subsequent studies of HRK were carried out using DP5, which shares 72% sequence similarity to human HRK and was identified in nerve growth factor withdrawal‐induced death of superior cervical ganglia neurons in rats [20]. DP5 is mainly expressed in brain tissue, with expression levels changing with mouse developmental stages [21]. Outside of its BH3 domain and C‐terminal TM region [21], the remaining HRK structure is less well characterised.

**Fig. 1:**
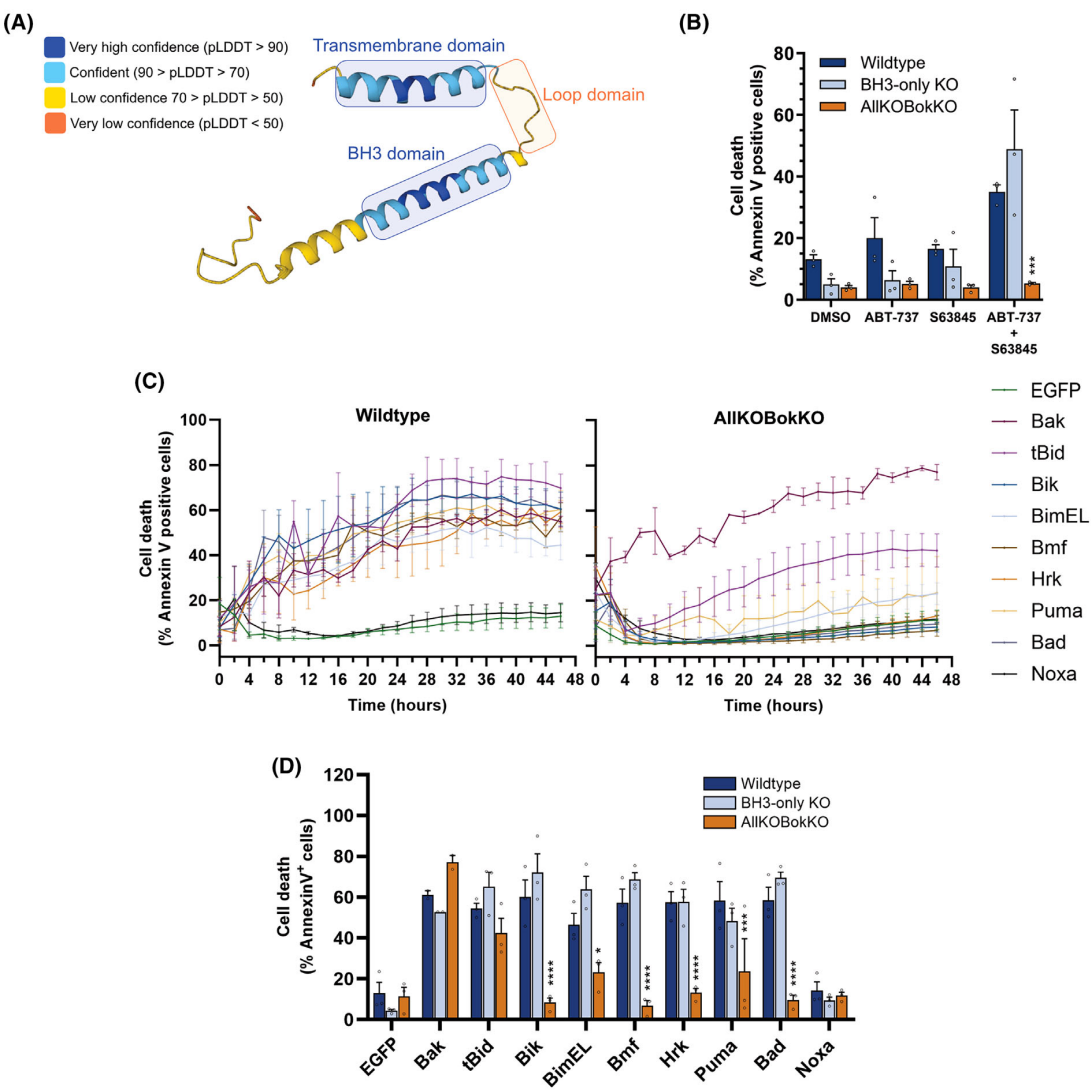
HRK‐induced cell death is dependent upon the presence of BAX or BAK. (A) alphafold prediction of the 3D structure of the Harakiri (HRK) protein. Figure reproduced from Refs [26, 27] with the addition of the highlighed protein domains. Per‐residue confidence scores produced by alphafold are depicted using the colour key. Key protein domains are outlined, with the region between the transmembrane domain and BCL‐2 homology (BH)3 domain denoted as the ‘loop domain’. (B) IncuCyte analysis of HCT116 wildtype, BH3‐only knockout (KO) and B cell lymphoma‐2 (BCL‐2) AllKOBokKO cells treated with either DMSO, ABT‐737, S63845 (1 μm) or a combination of ABT‐737 and S63845. Cell death quantification after 48 h of treatment, with dead cells analysed via Annexin V‐positive staining using incucyte analysis software. (C) Cell death time course of WT and BCL‐2 AllKOBokKO cells transfected with enhanced green fluorescent protein (EGFP)‐tagged BCL‐2 plasmids for 48 h. Dead cells were identified via Annexin V‐positive staining. (D) Comparison of Annexin V‐positive cells at the 48‐h treatment timepoint in panel (C). For all IncuCyte experiments, two wells were analysed per condition, with three representative images from each well used. All data were analysed using ANOVA comparing WT to BH3‐only KO or BCL‐2 AllKOBokKO values. Error bars represent standard error of three independent biological experiments. * = *P* < 0.05, *** = *P* < 0.005, **** = *P* < 0.001.

Since its discovery, only a handful of structural studies have been performed on HRK, in part due to recombinant forms of the protein proving toxic. Its BH3 domain is required for cell death induction [15], whilst the TM domain‐spanning residues are less well characterised. The first study to analyse the C‐terminal structure of HRK suggested that residues L65‐N91 are required for its localisation at intracellular membranes [22]. A later study by Barrera‐Vilarmau *et al*. [23] using truncated forms of HRK showed that, whilst the BH3 domain was necessary for binding to BCL‐XL and BCL‐2, its C terminus consisting of residues A61‐N91 was not required, but unknown regions upstream of the TM domain itself did improve binding strength. This is in partial agreement with an earlier study by Sunayama *et al*. [24] which agreed that the BH3 domain was required for binding to BCL‐XL, and a downstream region was required for binding to proteins outside of the BCL‐2 family, in this case the mitochondrial complement component 1 Q subcomponent‐binding protein, p32 (C1QBP). More recent studies have since predicted that a smaller range of residues may comprise the transmembrane region of HRK's C‐terminal domain, namely Y68‐R88 forming the transmembrane alpha‐helix [25]. Therefore, the precise residues comprising the TM domain, and if or how this domain or surrounding regions are required for cell death induction has not yet been fully established (Fig. 1A).

Under which conditions HRK is specifically involved in inducing cell death is not fully clear. Earlier studies investigating HRK/DP5‐induced cell death showed it plays a crucial role in regulating cell death in various neuronal cell types during growth factor withdrawal (superior cervical ganglion neurons [20] and haematopoietic progenitor cells [28]), in response to amyloid‐β protein (cortical neurons) [19], in low potassium conditions (cerebellar granule neurons) [16] and at different embryonic and developmental stages [21, 29]. Levels of HRK have been shown to be regulated at a transcriptional level via c‐Jun N‐terminal kinase (JNK) signalling [16] and the downstream regulatory element antagonist modulator (DREAM) calcium‐binding protein [30], but the specific mechanisms in terms of its regulation during and outside of apoptosis have yet to be fully explored. Furthermore, potential alternative functions of HRK have yet to be studied.

Here we show that HRK can induce cell death in the absence of other BH3‐only proteins, and whilst the BH3 domain of HRK is crucial for cell death induction, its TM domain is not. The TM domain localises HRK to the MOM where, when highly expressed in the absence of other BCL‐2 proteins, it induces mitochondrial aggregation. Intriguingly, the HRK TM domain alone is insufficient to target HRK to mitochondria, suggesting other areas within its structure are also responsible for both mitochondrial localisation and reorganisation independently of the BH3 domain and of cell death.

## Results

### HRK‐induced cell death is dependent only upon effector BCL‐2 proteins

We initially wanted to determine the importance of the HRK BH3 and TM domains in initiating cell death depending on the presence of other BCL‐2 family members. To measure cell death in a high‐throughput manner, we used IncuCyte live cell imaging analysis with Annexin V‐positive staining as a readout for cell death. First, we employed wildtype (WT) HCT116 cells and two BCL‐2 protein knockout lines, namely HCT116 BH3‐only KO, which have the main eight BH3‐only proteins removed (BAD, BID, BCL‐2‐interacting mediator of cell death (BIM), BCL‐2‐interacting killer (BIK), BCL‐2‐modyfing factor (BMF), HRK, NOXA and p53‐upregulated mediator of apoptosis (PUMA)), and the BCL‐2 AllKO cell line, which has the aforementioned BH3‐only proteins removed [31], as well as the anti‐apoptotic and effector proteins (including BOK [32]), hereafter termed ‘AllKOBokKO’. As expected, both WT and BH3‐only KO lines were sensitive to BAX and BAK‐induced death upon combination treatment of the BH3‐mimetics ABT‐737 (BCL‐XL and BCL‐2 inhibitor) and S63845 (MCL‐1 inhibitor) (Fig. 1B). This suggests the inhibition of anti‐apoptotic proteins is sufficient to induce BAX or BAK activation also in the absence of BH3‐only proteins. As expected, the BCL‐2 AllKOBokKO cell line was completely resistant to mimetic‐induced death due to the absence of effector proteins.

To compare the effects of BH3‐only proteins in regulating cell death, WT HCT116 cells were transiently transfected separately with plasmids containing coding sequences for N‐terminal enhanced green fluorescence protein (EGFP)‐tagged BH3‐only proteins BAD, BIK, BIM_EL_, BMF, NOXA, PUMA and HRK, or C‐terminal tagged tBID, and cell death was measured over a period of 48 h using Annexin V as a death marker (Fig. 1C, left panel). EGFP‐BAK and EGFP were used as positive and negative controls for cell death, respectively. In WT cells, BAK and all the individual BH3‐only proteins induced cell death in ~ 60% of the cell population, except NOXA, which induced cell death in a similar proportion of cells as EGFP‐transfected cells. BAD and NOXA are more selective in their binding affinities for anti‐apoptotic proteins, and as such are expected to kill fewer cells than other BH3‐only proteins [8]. Previous studies from our laboratory [32] and others [33] have shown that in the absence of other effector BCL‐2 proteins, tBID can act as an effector‐like protein to induce apoptosis. To confirm that only tBID is capable of this, BCL‐2 AllKOBokKO cells were transfected with the same set of BH3‐only proteins, and cell death was quantified. Indeed, no other BH3‐only proteins could induce cell death apart from tBID (Fig. 1C, right panel), and this was not due to higher expression levels of tBID compared to the other BH3‐only proteins (Fig. S1). With regard to HRK, it did not require the presence of other BH3‐only proteins to induce cell death, as cell death induction occurred in a similar proportion of cells in both HRK‐transfected WT and BH3‐only KO cells. Furthermore, BCL‐2 AllKOBokKO cells did not die upon HRK overexpression, underscoring the need for BAX or BAK for HRK‐induced cell death (Fig. 1D).

### HRK overexpression causes mitochondrial aggregation in the absence of other BCL‐2 proteins

**Fig. 2:**
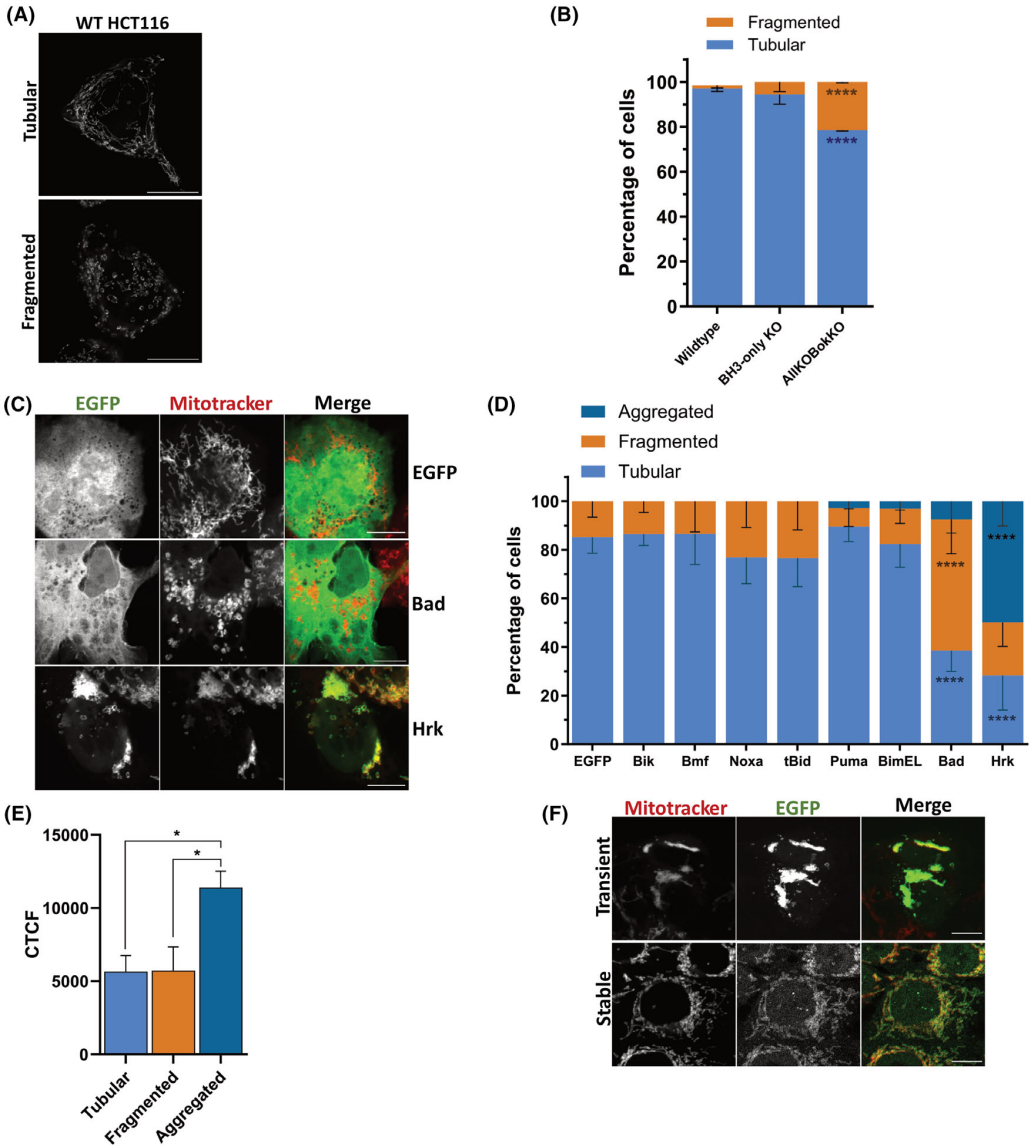
HRK overexpression causes mitochondrial aggregation that requires its transmembrane domain. (A) Confocal Airyscan immunofluorescence images of HCT116 wildtype (WT) cells stained with MitoTracker. (B) HCT116 WT, BCL‐2 homology (BH)3‐only knockout (KO) and BCL‐2 AllKOBokKO cells were stained with MitoTracker, fixed, and imaged via confocal microscopy to visualise mitochondrial morphology. Cells were categorised as having either tubular or fragmented mitochondria. Data represent *n* = 150 to 200 cells from representative fields of view, from three independent experiments. (C) Representative images of HCT116 BCL‐2 AllKOBokKO cells transfected with either enhanced green fluorescent protein (EGFP), EGFP‐Harakiri (HRK) or EGFP‐BCL‐2 antagonist of cell death (BAD) plasmids and stained with MitoTracker. (D) Mitochondrial morphology quantification of cells transfected with individual EGFP‐tagged BH3‐only protein plasmids. Data represent *n* = 75 to 100 cells from representative fields of view, from three independent experiments. (E) Quantification of corrected total cell fluorescence (CTCF) of EGFP‐positive cells analysed in (D) comparing EGFP intensity between cells with tubular, fragmented, or aggregated mitochondria. (F) Representative images of HCT116 BCL‐2 AllKOBokKO cells either stably or transiently expressing EGFP‐HRK and stained with MitoTracker for quantification of mitochondrial morphology. Data represent *n* = 150 to 200 cells from representative fields of view, from three independent experiments. All scale bars represent 10 μm. Images were analysed using fiji imagej. All data were analysed using ANOVA, comparing to WT cells (1B), EGFP (1D), or aggregated mitochondria (1E). Error bars represent the standard deviation of three independent biological replicates. * = *P* < 0.05, **** = *P* < 0.001.

Once confirmed HRK was incapable of acting as an effector‐like protein in the absence of other BCL‐2 proteins, we next wanted to examine HRK localisation in these cells, and if it had any involvement in regulating mitochondrial dynamics independently of other BCL‐2 proteins. HRK localisation has been previously visualised in the cytosol and at intracellular membranes [15], specifically the MOM [24, 34]. As other BCL‐2 proteins such as BAX and BAK [35], BOK [36], BCL‐XL [37], and BID [38] have been implicated in regulating mitochondrial morphology, we also wanted to examine if HRK also contributes to this function. We began by comparing mitochondrial morphology between HCT116 WT, BH3‐only KO, and BCL‐2 AllKOBokKO cells using MitoTracker staining to determine if removing families of BCL‐2 proteins altered the morphology. Following fixation, cells were imaged via confocal microscopy. We classified mitochondria into two distinct phenotypes: tubular and fragmented. Representative images illustrating these mitochondrial phenotypes in WT cells are shown in Fig. 2A. The majority of WT and BH3‐only KO cells exhibited tubular mitochondria, with a small percentage containing more fragmented mitochondria. Notably, the BCL‐2 AllKOBokKO cells had a significantly larger proportion of cells with fragmented mitochondria than WT cells (Fig. 2B). This may be explained by the lack of BAX and BAK in these cells, as these proteins have been shown to be required for the normal fusion of mitochondria in healthy cells [35].

To examine the localisation and effect of individual BH3‐only proteins on mitochondria, all the BH3‐only plasmids examined in Fig. 1C,D were transfected into HCT116 BCL‐2 AllKOBokKO cells, and mitochondria labelled using MitoTracker Deep Red 24 h after transfection. Cells were fixed and imaged to analyse BH3‐only protein localisation and mitochondrial morphology (Fig. 2C and Fig. S2A). Interestingly, whilst overexpression of most of the proteins did not alter mitochondrial morphology in comparison to EGFP (Fig. S2A), BAD and HRK showed distinctly altered mitochondrial shapes (Fig. 2C). BAD induced mitochondrial fragmentation in approximately 50% of cells, including numerous cells containing small, circular (or ‘donut’) shaped mitochondria, whereas HRK caused significant mitochondrial aggregation in 50% of cells (Fig. 2D). Furthermore, BAD did not localise to mitochondria, appearing more ER‐localised, whereas HRK localised almost exclusively to mitochondria. The changes in morphology did not appear to be related to differing expression levels between plasmids, as quantification of EGFP expression of each BH3‐only protein showed variation, but no significant differences (Fig. S2B). However, the EGFP fluorescence intensities of HRK between cells with tubular or fragmented mitochondria versus aggregated mitochondria differed, with cells with aggregated mitochondria having on average a higher EGFP fluorescence intensity (Fig. 2E). Using lentiviral transduction, we created a HCT116 BCL‐2 AllKOBokKO cell line stably expressing EGFP‐HRK (termed AllKOBokKO‐EGFP‐HRK) and analysed HRK localisation and mitochondrial morphology in these cells. Interestingly, we only obtained cells with lower expression levels of HRK, suggesting that high levels of the protein are not well tolerated by cells. Whilst HRK was again localised mainly at the MOM, the morphology of the mitochondria appeared mainly tubular with lower overall EGFP expression (Fig. 2F). Together, these data suggest that HRK localises to mitochondria and can induce mitochondrial network reorganisation upon high levels of expression.

### HRK overexpression does not alter inner mitochondrial morphology or respiration

**Fig. 3:**
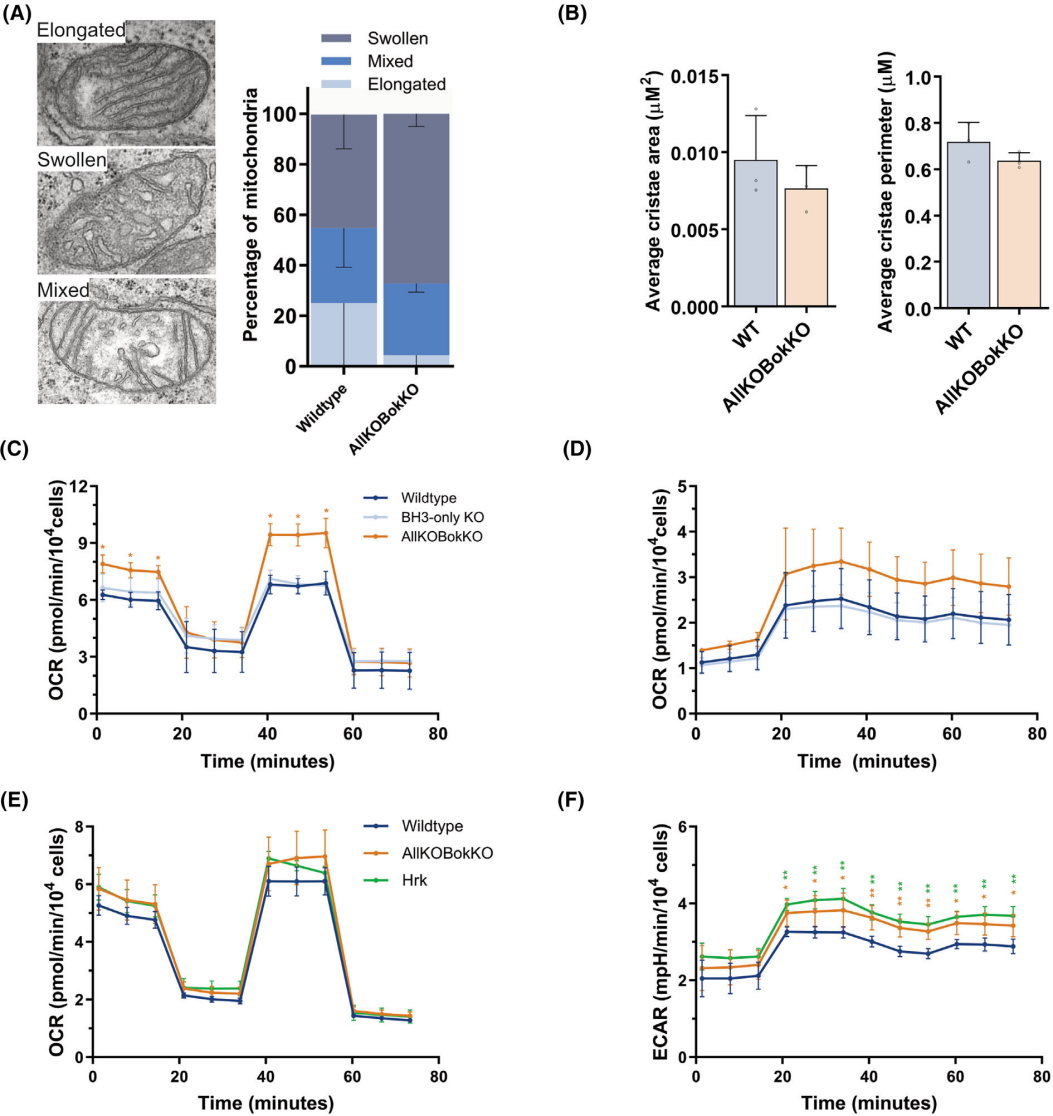
HRK overexpression does not alter inner mitochondrial morphology or respiration. (A) Representative transmission electron tomography images of mitochondria from HCT116 cells demonstrating elongated, swollen, or mixed morphologies with quantification of morphology distribution in wildtype (WT) and HCT116 AllKOBokKO cells. (B) Analysis of cristae area and perimeter from images as in (A). Data represent *n* = 80 to 100 mitochondria from three independent experiments. (C) Seahorse mitochondrial stress test analysis of oxidative phosphorylation (OXPHOS) showing WT, BCL‐2 homology (BH)3‐only knockout (KO) and AllKOBokKO oxygen consumption rate (OCR). (D) Glycolysis analysis from the same dataset as in (C). (E) Seahorse mitochondrial stress test of WT, AllKOBokKO, and AllKOBokKO‐EGFP‐HRK cells, showing OXPHOS measurements. (F) Glycolysis analysis as in (E). All data were analysed using ANOVA, comparing to WT cells. Error bars represent standard deviation of three independent experiments. * = *P* < 0.05, ** = *P* < 0.01 comparing all values to WT cells.

We next compared mitochondrial inner membrane (MIM) morphology of WT and BCL‐2 AllKOBokKO HCT116 cells via transmission electron microscopy (TEM). Three distinct morphologies of cristae were visualised in the cell lines—elongated, swollen and mitochondria containing a mix of both elongated and swollen cristae (Fig. 3A). In both cell lines, approximately 50% of cristae appeared swollen, with approximately 10% elongated. This phenotype was also observed in live cells via STED microscopy using the MIM dye PKMito Orange FX [39] (Fig. S3A). Quantification of cristae structure revealed that, whilst a mix of cristae morphologies was visible, the average cristae area and perimeter were comparable between both cell lines (Fig. 3B), suggesting BCL‐2 proteins do not play a significant role in regulating cristae structure. Imaging of the BCL‐2 AllKOBokKO‐EGFP‐HRK cells also revealed no significant differences to the parental KO line (Fig. S3B), showing HRK re‐expression does not modulate cristae structure.

BCL‐2 proteins have been implicated in the regulation of additional mitochondrial functions, including regulating mitochondria‐ER contact sites (MERCS). MERCS are regions within cells where mitochondria and ER are in close proximity (Fig. S3C), acting as signalling hubs for a multitude of metabolic processes including regulating calcium signalling [40]. Whilst anti‐apoptotic proteins are known to play significant roles in this process, BH3‐only proteins could potentially also be involved. Therefore, using the same TEM images as previously, we analysed whether there were any differences in MERCS between WT and BCL‐2 AllKOBokKO cell lines (Fig. S3B). There were no significant differences in either MERC contact length or distance between the two cell lines. Cells stably overexpressing HRK also showed no difference in MERC distance or contact length (Fig. S3B), thus HRK mitochondrial localisation is not involved in regulating MERC formation or structure.

Finally, we wanted to determine if HRK overexpression altered cellular respiration. Previous studies have proposed roles for BCL‐XL, BCL‐2 and MCL1 in increasing mitochondrial respiration [41, 42, 43, 44], and BAX and BAK in decreasing mitochondrial respiration [42, 45]. The roles of BH3‐only proteins are less well studied, with varying results seen for BIM [46], PUMA [47] and BAD [10], but no studies have examined HRK. We began by subjecting HCT116 WT, BH3‐only KO and BCL‐2 AllKOBokKO cells to Seahorse respiration analysis of oxygen consumption rate (OCR) and extracellular acidification rate (ECAR) for oxidative phosphorylation (OXPHOS) and glycolysis, respectively. Whilst there were no significant differences between WT and BH3‐only KO cells, the BCL‐2 AllKOBokKO cells had significantly higher basal, maximal and ATP‐linked respiration rates (Fig. 3C). The AllKOBokKO cells also had generally higher rates of glycolysis, though this was not statistically significant due to greater variability in ECAR measurements (Fig. 3D). These results indicate that the BCL‐2 family of proteins play a role in regulating OXPHOS in HCT116 cells.

We then subjected the HCT116 AllKOBokKO‐EGFP‐HRK cells to the same mitochondrial stress test and compared OCR and ECAR values with WT and AllKOBokKO cells. Here, we found no difference in OCR between AllKOBokKO‐EGFP‐HRK cells and AllKOBokKO or WT cells (Fig. 3E), although the maximal respiration rate of AllKOBokKO cells was overall lower compared to previous experiments (Fig. 3C). ECAR measurements revealed that both the AllKOBokKO cells and AllKOBokKO‐EGFP‐HRK cells had significantly higher glycolysis rates than the WT cells, but with no difference between both KO cell lines (Fig. 3F). Taken together, these data suggest that, whilst HRK is capable of localising to mitochondria and affecting their morphology, it does not affect MIM structure or respiration in doing so.

### HRK‐induced cell death is BH3 domain‐dependent, but not transmembrane‐domain‐dependent

**Fig. 4:**
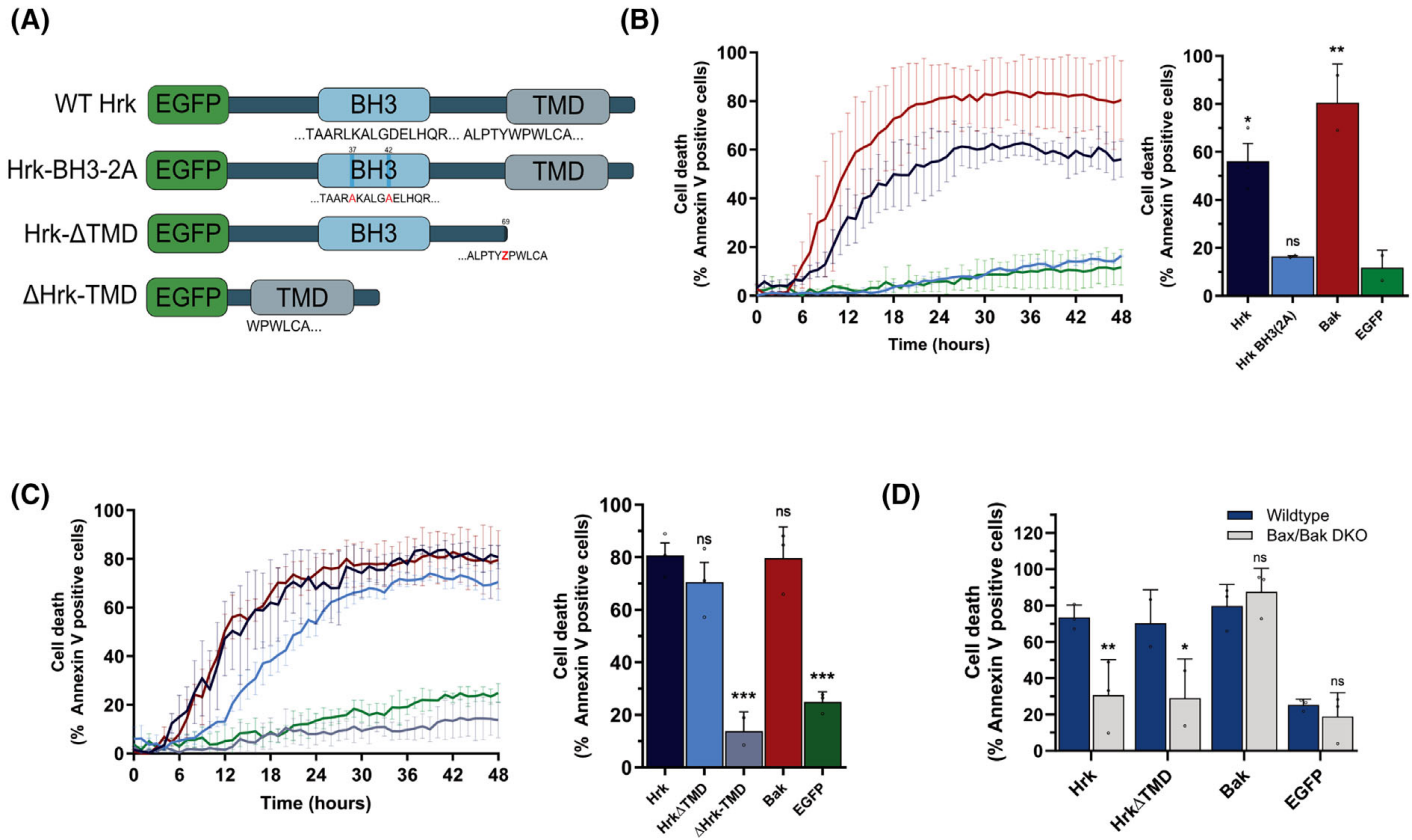
HRK‐induced cell death is BH3 domain, but not transmembrane‐domain‐dependent. (A) Schematic of Harakiri (HRK) mutant plasmids created for functional analysis. Red letters indicate substituted residues. (B) IncuCyte cell death assay of wildtype (WT) HCT116 cells transfected with either WT HRK, HRK‐BCL‐2 homology (BH)3(2A), BCL‐2 antagonist/killer 1 (BAK) or enhanced green fluorescent protein (EGFP), and cell death analysed over 48 h. Left hand panel shows the 48‐h time course; right hand panel shows the comparison of cell death after 48 h of transfection. Data represent *n* = 3 independent experiments. (C) incucyte analysis as in B of cells transfected with either EGFP‐tagged WT HRK, HRKΔTMD (HRK lacking its transmembrane domain), ΔHRK‐TMD (only the TMD of HRK), BAK or EGFP and cell death analysed over 48 h. Left hand panel shows the 48‐h time course; right hand panel shows the comparison of cell death after 48 h of transfection. Data represent *n* = 4 independent experiments. (D) incucyte cell death analysis as in (C) of HCT116 WT or BCL‐2 associated X, apoptosis regulator (BAX)/BAK double‐knockout (DKO) cells transfected with EGFP‐tagged WT HRK, HRK‐ΔTMD, BAK, or EGFP for 48 h. Data were statistically analysed by ANOVA comparing DKO values to WT. For all incucyte experiments, two wells were analysed per condition, with three representative images from each well used. All data were analysed using ANOVA, comparing to EGFP (B), HRK (C), or WT cells (D). Error bars represent standard deviation of three independent experiments. * = *P* < 0.05, ** = *P* < 0.01, *** = *P* < 0.005.

Taking the results observed so far, that HRK promotes BAX/BAK‐dependent cell death and can induce mitochondrial aggregation, we next wanted to dissect the molecular mechanisms involved. To this aim, we investigated the importance of both the C‐terminal TM domain and the BH3 domain in these processes. We therefore created three N‐terminal EGFP‐tagged HRK mutants: one containing two alanine substitution mutations in key BH3 domain residues, L37 and D42 [19] (termed HRK‐BH3(2A)); HRK lacking its TM domain due to the insertion of a premature stop codon at the beginning of the alpha‐helical region within its TM domain coding sequence at W69 (HRK‐ΔTMD) based on the predicted TMD region outlined in Nguyen *et al*. [25] (specifically, lacking the membrane‐inserting alpha‐helix from W69‐L85); a construct expressing EGFP upstream of the HRK TM sequence alone from W69 onwards (ΔHRK‐TMD) (Fig. 4A).

As previous studies have demonstrated the requirement of the BH3 domain of HRK in interacting with anti‐apoptotic BCL‐2 proteins [15, 24, 48], we first examined the effect of mutating key BH3 domain residues in the HRK BH3 domain on cell death in HCT116 cells. WT HCT116 cells were transfected with plasmids encoding either WT HRK, HRK BH3(2A), BAK or EGFP, and cell death was analysed using the IncuCyte as previously. As expected, both BAK and WT HRK induced cell death in a significant proportion of cells, with EGFP inducing minimal amounts (Fig. 4B). HRK BH3(2A) induced a comparable percentage of cell death to EGFP, showing the requirement of a functioning BH3 domain to induce apoptosis. The presence of endogenous HRK or other BH3‐only proteins did not affect the proportion of cell death observed, as similar results were obtained in BH3‐only KO cells (Fig. S4A).

Next, we analysed the requirement of the HRK TM domain in HRK‐induced cell death by transfecting HCT116 WT cells with either EGFP‐WT HRK, HRK‐ΔTMD or ΔHRK‐TMD plasmids and measuring Annexin V staining via IncuCyte imaging. Interestingly, whilst the TM domain alone (ΔHRK‐TMD) was unable to induce apoptosis, most likely due to a complete lack of the BH3 domain, HRK‐ΔTMD induced cell death in a similar proportion of cells as both WT HRK and BAK (Fig. 4C). As the proportion of the cell population killed by HRK‐ΔTMD was slightly, but not significantly lower than WT HRK, we titrated levels of HRK and HRK‐ΔTMD plasmids to assess whether, at lower expression levels, the TMD mutant would still be as potent at killing the HCT116 cells (Fig. S4D). Although transfection efficiency was significantly lower at lower plasmid concentrations (~ 20% at 100 ng, ~ 5% at 50 ng and ~ 1% at 25 ng), the HRK‐ΔTMD mutants did still induce cell death, but at an impaired level compared to WT HRK. The reduced killing was not due to a lower expression of the mutants; on the contrary, in the cells transfected with 100 ng of plasmid, the HRK‐ΔTMD cells had slightly increased EGFP expression (Fig. S4E). Therefore, whilst the HRK TMD is not required for cell death, its presence increases the potency of its pro‐death activity. Furthermore, the rate of cell death in HRK‐ΔTMD‐transfected cells appeared reduced. This data is in line with previous studies showing that HRK lacking its TM domain can still bind to (and therefore sequester) anti‐apoptotic BCL‐2 proteins, although potentially with a lower affinity [48] which could account for the lower rates of cell death induction seen here. Similar to the HRK‐BH3(2A) experiments, the presence of endogenous BH3‐only proteins did not affect this interaction as HCT116 BH3‐only KO cells displayed similar proportions and kinetics of cell death to the WT cells (Fig. S4B,C). The cell death was also BAX/BAK dependent, as the proportion of Annexin V‐positive cells expressing HRK‐ΔTMD was similar to EGFP‐transfected cells in BAX/BAK DKO HCT116 cells (Fig. 4D). Therefore, whilst HRK requires its BH3 domain to induce cell death, it does not require a TM domain nor mitochondrial localisation.

### HRK requires sequences upstream of its transmembrane domain to localise to mitochondria

**Fig. 5:**
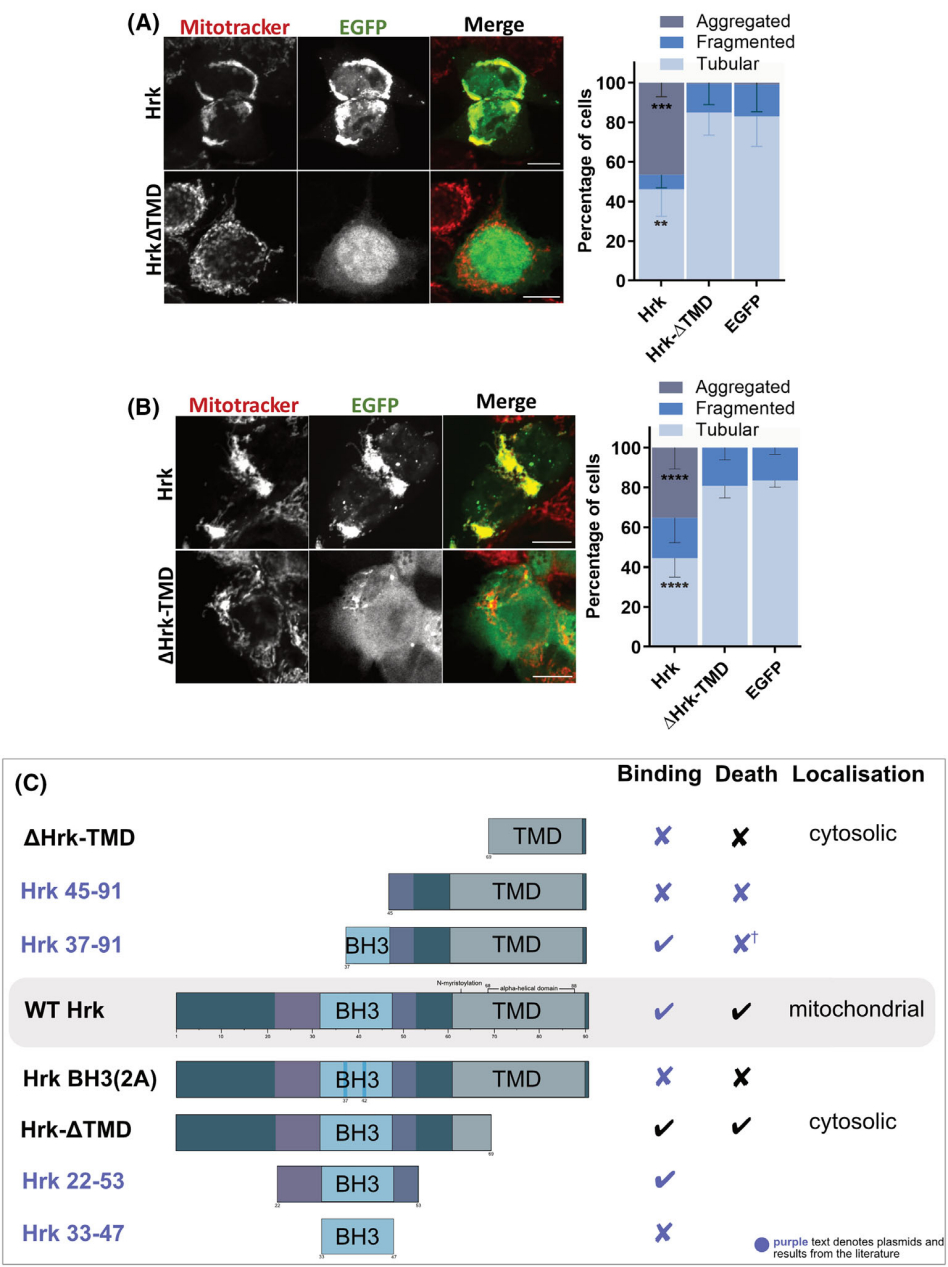
HRK transmembrane domain is insufficient to target to mitochondria. Confocal immunofluorescence images and quantification of HCT116 B cell lymphoma‐2 (BCL‐2) AllKOBokKO cells transiently transfected with either enhanced green fluorescent protein (EGFP)‐Harakiri (HRK) or EGFP‐HRKΔTMD (HRK lacking its transmembrane domain) plasmids and stained with MitoTracker. Data represent *n* = 100 to 125 cells from representative fields of view from three independent experiments. (B) Confocal immunofluorescence images and quantification of HCT116 AllKOBokKO cells transiently transfected with either EGFP‐HRK or EGFP‐ΔHRK‐TMD plasmids and stained with MitoTracker. Data represent *n* = 100 to 125 cells from representative fields of view from three independent experiments. (C) Schematic of HRK structures that have been studied here and elsewhere in the literature, and indication of whether the constructs have been experimentally shown to be capable of inducing cell death (“Death”), involved in binding interactions with other BCL‐2 family/apoptosis‐related proteins (“Binding”), and where they have been visualised within the cell (“Localisation”). Purple text denotes information from elsewhere in the literature. † denotes unpublished data. See References [20, 21, 45] for the original studies that used the constructs. Scale bars represent 10 μm. All data were analysed using ANOVA comparing to EGFP. Error bars represent standard deviation of three independent experiments. ** = *P* < 0.01, *** = *P* < 0.005, **** = *P* < 0.001.

As the TM domain of HRK was not required for cell death induction, we wanted to determine if the TM domain was required for induction of mitochondrial network rearrangement. We transfected HCT116 BCL‐2 AllKOBokKO cells with either WT HRK, HRK‐ΔTMD, or EGFP plasmids and analysed mitochondrial morphology using MitoTracker as before. Unlike WT HRK, HRK‐ΔTMD did not induce mitochondrial aggregation, with comparable morphologies to EGFP‐transfected cells (Fig. 5A). The EGFP signal was also no longer localised to mitochondria, with distribution between the cytosol and nucleus. Thus, HRK requires its TM domain to localise to mitochondria and induce mitochondrial aggregation when transiently overexpressed. To determine if the TM domain of HRK alone is capable of altering mitochondrial morphology, we repeated the previous experiment, this time transfecting ΔHRK‐TMD (Fig. 5B). Analysis of mitochondrial morphology indicated that the EGFP‐tagged TM domain of HRK alone does not induce mitochondrial aggregation. Surprisingly, the ΔHRK‐TMD protein also did not localise to mitochondria, showing a mainly cytosolic distribution. This suggests that the HRK TM domain alone is insufficient to target to mitochondria, requiring other domains of the protein.

Combining the results from our analysis with those previously found in the literature, we can begin to create a clearer picture of how the structure of HRK influences its function (Fig. 5C), with the residues comprising the minimal required mitochondrial‐targeting span within the TM domain becoming clearer.

## Discussion

Here we show that the BH3‐only protein HRK can induce apoptosis via a BH3‐domain‐dependent, but not TM domain‐dependent mechanism. The TM domain localises HRK to the MOM where HRK can alter mitochondrial structure with little effect on mitochondrial respiration. However, the TM domain alone is insufficient for mitochondrial targeting, requiring additional sequences within the HRK structure.

Whilst the ability of HRK to induce mitochondrial aggregation appears to be dependent on its overexpression, it is interesting that none of the other BH3‐only proteins—even those localised to mitochondria—caused the same effect when overexpressed, with only BAD causing fragmented mitochondria. These results set HRK apart from other BH3‐only proteins and suggest that its localisation at the MOM may have additional roles in aggregating mitochondria prior to cell death when death is delayed, or an additional non‐apoptotic function.

Since HRK can affect the organisation of the mitochondrial network, we hypothesised that overexpression of HRK may alter MIM structure or metabolism in the HCT116 cells. Stable expression of HRK in BCL‐2 AllKOBokKO cells, however, had no significant effect on cristae structure. Analysis of OXPHOS and glycolysis via Seahorse also identified no significant changes in respiration between BCL‐2 AllKOBokKO cells and those stably expressing EGFP‐HRK, suggesting that, at least in cells lacking other BCL‐2 proteins, HRK does not regulate respiration. It is important to note that the maximal respiration level reached by the AllKOBokKO cells was lower in the experiments including the stable AllKOBokKO‐HRK line (Fig. 3E) compared to those comparing the HCT116 KO lines (Fig. 3C). This is likely caused by slight variations in cell numbers due to cells detaching during the injection and mixing points of assay, as well as the AllKOBokKO and HRK stable lines being a higher passage due to the growth time required to create the stable cell lines. Nonetheless, HRK may have a potential functional role in metabolic regulation via its interaction with anti‐apoptotic BCL‐2 proteins known to regulate OXPHOS. For example, BCL‐XL decreases glycolysis [41] whereas BAX has been shown to decrease mitochondrial respiration, which can be antagonised by BCL‐XL [42]. In our mitochondrial stress test, BCL‐2 AllKOBokKO cells had both increased OXPHOS and glycolysis compared to WT, which was not affected by re‐expression of HRK. One could hypothesise that, as HRK is a known interactor with BCL‐XL, it requires the presence of it, or indeed other BCL‐2 family proteins to modulate respiration or other similar mitochondrial functions. Further examination of these assays with cells containing BCL‐2 family proteins will be required to determine the functional role of HRK at the MOM.

Within the BCL‐2 protein family, anti‐apoptotic and effector proteins are similar in structure, with TM domains that localise the proteins to lipid membranes, and in certain instances, the TM domains alone are sufficient for this localisation [49]. Indeed, swapping the TM domains of BCL‐XL and BCL‐2, which mainly localise to the MOM and ER, respectively, causes the recipient protein to localise to membrane compartments of the TM domain parent protein [50]. BH3‐only proteins are simpler in structure than the multi‐domain proteins, containing only one BH3 domain and, for some, including Bik and HRK, a TM domain. Previous studies examining the structure of HRK have identified the sequence of its C‐terminal domain, although with some ambiguity over the precise location of the membrane‐spanning sequence. When first discovered, HRK was predicted to have a putative TM domain spanning residues P60‐L86 based on comparisons with other known BCL‐2 family proteins [15]. Studies have since suggested the TM domain is further towards the C‐terminus; Barrera‐Vilarmau *et al*. [23] studied residues A61‐N91 when analysing a HRK mutant spanning the TM domain; whereas Bernabeu *et al*. [22] identified a smaller spanning region of residues, L65–L91, by plotting average surface hydrophobic moments, hydrophobicity, and interfaciality. More recently, using the web server transmembrane predictor PSIPRED [51], the helix‐forming amino acid sequence within HRK's C‐terminal region was predicted to span a smaller number of residues, Y68‐R88 [25], with information on UniProt suggesting this minimal TMD span may be as small as W69‐G87 (UniProt ID: O00198). In this study, we therefore created HRK TMD mutants using tryptophan 69 as the first residue of the TM domain to examine as small a span as necessary.

Sensitiser proteins do not necessarily require MOM localisation capabilities or a membrane‐spanning domain to sequester apoptosis‐regulating proteins like BCL‐XL and DIVA [48], thus instigating the question why some such as HRK have a membrane targeting sequence within their structure at all. The TM region could be involved in promoting strong binding to anti‐apoptotic BCL‐2 proteins; as demonstrated by the Andrews lab, some BH3‐only proteins like BIM and PUMA can bind to the hydrophobic pocket of anti‐apoptotic proteins using a ‘double bolt lock’ system consisting of key residues in the BH3 domain, as well as residues within their TM domains [52, 53]. This creates a higher affinity interaction than would be achieved with BH3 domain binding alone. As HRK is classed as an apoptotic sensitiser, having a tight bond with anti‐apoptotic proteins is likely beneficial in sequestering anti‐apoptotic BCL‐2 proteins when cells are targeted for death.

As an analysis of different mutant forms of HRK showed, whilst HRK requires its TM domain for localisation at mitochondria, the TM domain is not required for induction of cell death. It does, however, increase the apoptotic potency of HRK, as removal of its TM domain decreases levels of cell death induction. This again suggests that HRK binds to anti‐apoptotic proteins via a double bolt lock mechanism, with stronger binding causing a higher level of anti‐apoptotic protein sequestration. Interestingly, the TM domain alone fused to EGFP did not localise to the MOM. This suggests that mitochondrial localisation requires both the TM domain and another, more N‐terminal region of the protein. An N‐myristoylation sequence, known to be important for membrane targeting of multiple proteins [54] is thought to be located at the C‐terminus of HRK at residue G63 [23]. The HRK‐ΔTMD mutant contained this residue but was truncated downstream, whereas the ΔHRK‐TMD plasmid was truncated upstream of this site (c.f. Fig. 4A). As neither plasmid localised to mitochondria, this suggests both the G63 myristoylation site and the C‐terminal region are required for mitochondrial targeting, highlighting this as a potentially crucial residue for HRK localisation.

Further analysis of the loop domain and potentially regions upstream of the BH3 domain need to be carried out to fully elucidate which domains are important. Based on the aforementioned studies, we can hypothesise a minimal structure required for HRK‐induced cell death (Fig. 5C), most likely at least spanning the full BH3 domain plus a short extra region downstream. For membrane localisation, it is likely the C‐terminal domain from the G36 myristoylation site onwards is required. Further mutational studies analysing these regions within HRK will be required to fully elucidate its membrane binding mechanism, coupled with further functional studies to fully determine what non‐apoptotic functions HRK regulates.

In summary, our study identifies domain‐dependent interactions within HRK with distinct functions in cell death and mitochondrial organisation. HRK requires a functional BH3 domain to induce cell death, but for which the TM domain or mitochondrial localisation is not required. The TM helix, in combination with other upstream domains of HRK, plays a role instead in localising the protein to the mitochondria. There, whilst HRK does not appear to alter respiration dynamics, it has the capability to alter mitochondrial morphology when overexpressed in the absence of other BCL‐2 proteins. The TM domain is not required for cell death but can increase the rate of this process most likely by aiding in binding with anti‐apoptotic proteins at the MOM. The TM domain alone is, however, insufficient to target HRK to mitochondria, and we speculate that the short region between the BH3 and TM domains may play a role in HRK mitochondrial localisation. The precise function of the mitochondrial shape reorganisation by HRK identified here, which does not affect respiration or MERCS, will require elucidation in future studies.

## Materials and methods

### Expression constructs

For WT constructs, BH3‐only gene sequences were cloned from donor plasmids into a pEGFP‐C3 backbone apart from BAK and tBID, which were in a pEGFP‐C1 and pEGFP‐N2 backbone, respectively. Donor plasmids were purchased from Addgene (Watertown, MA, USA) (pHA‐PUMA, pEGFP‐Bik, pCDNA3 T7 Bmf, pGEX 4T1 hBad, pLenti CMV TRE3G BCL2L11 Neo, pLVX‐AcGFP‐C1). The WT NOXA plasmid was generously gifted by Andreas Villunger (Institute for Developmental Immunology, Biomedical University of Innsbruck). The plasmids encoding EGFP‐tagged BAK and tBID were previously generated and described in [32, 55]. HRK mutant plasmids were generated using site‐directed mutagenesis from the pEGFP‐C3 WT plasmid: HRK‐BH3(2A) (L37A and D42A) and HRK‐ΔTMD (W69Z). ΔHRK‐TMD was generated by cloning the TM domain from residue W69 onwards into pEGFP‐C3. For AllKOBokKO‐EGFP‐HRK stable cell line generation, lentivirus expression vectors were generated by cloning the EGFP‐HRK sequence into pENTR11. EGFP‐HRK was then cloned from pENTR11 into pLenti CMV Puro DEST, creating pLenti‐EGFP‐HRK.

### Cell culture

HCT116 WT (DSMZ, Braunschweig, Germany; RRID: CVCL_0291), HCT116 BAX/BAK DKO [56], HCT116 BCL‐2 AllKOBokKO [32], and HCT116 BH3‐only KO [31] cells were cultured in McCoy's 5A medium (Gibco, Waltham, MA, USA). HEK293T cells (DSMZ; RRID: CVCL_0063) were cultured in high‐glucose DMEM (Gibco). Media were supplemented with 10% FBS (PAN‐Biotech, Aidenbach, Germany) and 1% penicillin/streptomycin (Bio&Sell, Nürnberg, Germany). All cell lines were maintained at 37 °C with 5% CO_2_ under humidified conditions and passaged when 70–80% confluent. To ensure cell line authenticity, WT cells were sourced from a reputable vendor, and KO cells were validated by western blot. Only mycoplasma‐negative cells, tested with the Mycoplasma Detection Kit (Invivogen, Toulouse, France), were used in experiments.

### Plasmid transfection

Cells were seeded at the appropriate density 48 h before transfection. On the day of transfection, plasmid DNA was diluted to ensure 100 ng DNA in 25 μL of Opti‐MEM™ (per well of a 96‐well plate; Thermo Fisher Scientific, Waltham, MA, USA) or 1000 ng DNA in 100 μL of Opti‐MEM™ (per well of a 12‐well plate). 300 ng of linear, 25 kDa polyethylenimine (Polysciences, Warrington, PA, USA) was diluted per 100 ng DNA in the same volume of Opti‐MEM™ as DNA and incubated for 5 min at room temperature. Diluted DNA and linear, 25 kDa polyethylenimine were then combined and incubated for a further 30 min. Previous growth medium was then removed from the target cells and replaced with fresh medium before adding the transfection mixture dropwise. Cells were incubated for 24 h before further assaying.

### Lentiviral transduction

For creation of lentivirus, psPAX2 and pMD2.G vectors were transfected into HEK293T cells along with pLenti‐EGFP‐HRK using Lipofectamine 2000 (Thermo Fisher Scientific) following the manufacturer's instructions. Transfected HEK293T cells were incubated for 48 h before collecting the supernatant for transferring to target HCT116 cells. Infected cells were cultured for 2 weeks before fluorescence‐activated cell sorting for EGFP‐positive cells.

### Cell death assays

Cells were seeded into 96‐well plates 48 h prior to transfection at a density of 2500 cells per well and transfected as outlined above. Directly prior to the addition of transfection complexes to the cells, the culture medium was removed from each well and replaced with fresh culture medium containing 0.01% (v/v) of Annexin V Red (Sartorius, Göttingen, Germany). Immediately after the addition of transfection complexes, the plate was transferred into the IncuCyte S3 (Sartorius) imaging system. Three images per well were acquired every hour for 48 h before analysing the total number of cells via the cell‐by‐cell incucyte analysis software. Percentage cell death was calculated by normalising the number of Annexin V‐positive cells to total transfected cells.

For cells treated with BH3‐mimetics, cells were seeded into 96‐well plates 48 h prior to treatment at a density of 2500 cells per well. On the day of treatment, fresh medium containing 0.01% (v/v) of Annexin V Red was added to each well, with either 1 μm of ABT‐737 (APExBIO, Houston, TX, USA) or S63845 (ActiveBiochem, San Jose, CA, USA), or DMSO (Thermo Fisher Scientific) as a control. Three images per well were acquired every hour for 48 h before analysing the total number of cells via the cell‐by‐cell incucyte analysis software.

### Seahorse mitochondrial stress test

The appropriate plating density for each cell line was optimised prior to assay setup—between 1500 and 2500 cells per well. Cells were seeded into Seahorse XFe96 well plates (Agilent, Santa Clara, CA, USA) 48 h prior to running the assay to ensure between 60% and 80% confluency. The mitochondrial stress test was performed on a XFe96 analyser (Agilent) using the Seahorse XF Cell Mito Stress kit (Agilent) following the manufacturer's instructions, optimising FCCP concentration prior to experimental setup. Cell number was normalised using pre‐assay brightfield imaging and post‐assay Hoechst 33342 (Thermo Fisher Scientific) staining via BioTek Cytation 5 (Agilent).

### Immunofluorescence microscopy

Cells were seeded onto coverslips in a 12‐well plate, 48 h before transfection, at a density of 50 000 cells per well. 24 h after transfection, cells were gently washed with PBS before incubating for 20 min with 150 nm of MitoTracker Deep Red FM (Invitrogen, Carlsbad, MA, USA) in fresh growth medium. Cells were then gently washed 3 times (5 min) with PBS before fixation with 4% formaldehyde (Thermo Fisher Scientific). Fixed cells were incubated with 1 μg·mL^−1^ of Hoechst 33342 for 5 min before three PBS washes (5 min). Cells were then washed once with H_2_O before leaving to dry overnight. Coverslips were then mounted using mounting medium and left to dry before imaging. Confocal imaging was carried out using the SM Meta 710 (Zeiss, Oberkochen, Germany) equipped with a Plan‐Apochromat 63×/1.4 Oil DIC (Zeiss) objective. Fluorescence intensity measurements were analysed using fiji [57]. Briefly, individual cells were outlined manually using the Freehand ROI tool, and area, integrated Density, and mean Grey Value measured. Background measurements were also taken in areas containing no cells. Corrected total cell fluorescence (CTCF) was calculated as Integrated Density − (Cell Area × background).

### Transmission electron tomography

Cells were seeded onto Aclar foils (VWR, Radnor, PA, USA) 48 h before fixation to ensure a minimum of 80% confluency. Cells were fixed in pre‐warmed fixative (4% formaldehyde with 2.5% sucrose (Sigma‐Aldrich, Burlington, MA, USA) and 100 mm CaCl_2_ (Sigma‐Aldrich) in HEPES (Thermo Fisher Scientific) buffer, pH 7.4) for 30 min at room temperature followed by 30 min at 4 °C. Foils were then washed with 0.1 m cacodylate buffer (Sigma‐Aldrich) before processing for imaging with the EM‐2100 Plus Transmission Electron Microscope (JEOL, Tokyo, Japan) operating at 80 kV equipped with a OneView 4 K camera (Gatan, Pleasanton, CA, USA). Mitochondria and cristae structure were analysed manually using fiji as outlined by Lam *et al*. [58].

## Conflict of interest

The authors declare no conflict of interest.

## Author contributions

LEK and AJG‐S conceptualised the study. LEK and LF performed and analysed experiments. LEK and LF wrote the manuscript.

## Supporting information


**Fig. S1.** tBID‐induced cell death in AllKOBokKO cells is not due to higher expression of tBID.
**Fig. S2.** Overexpression of BH3‐only proteins other than HRK and BAD do not alter mitochondrial morphology.
**Fig. S3.** HRK overexpression does not alter MERCs.
**Fig. S4.** BH3‐only proteins do not affect HRK‐induced cell death.

## Data Availability

The data that support the findings of this study are available from the corresponding author (ana.garcia@biophys.mpg.de) upon reasonable request.
