# Supplementary material for: Activator of apoptosis harakiri (HRK) localisation at mitochondria alters mitochondrial morphology independently of other BCL‐2 proteins
Source: FEBS J. 2025 Sep 12;293(2):549–64. doi: 10.1111/febs.70255 (PMC12820597; doi:10.1111/febs.70255)
Supplement: Supplementary file 1 — Fig. S1. tBID‐induced cell death in AllKOBokKO cells is not due to higher expression of tBID. Fig. S2. Overexpression of BH3‐only proteins other than HRK and BAD do not alter mitochondrial morphology. Fig. S3. HRK overexpression does not alter MERCs. Fig. S4. BH3‐only proteins do not affect HRK‐induced cell death. [file FEBS-293-549-s001.pdf]

Supplementary Figures

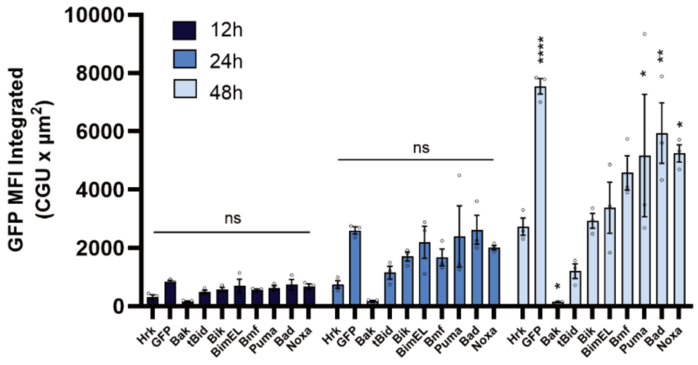

Figure S1 – tBID induced cell death in AllKOBokKO cells is not due to higher expression of tBID. HCT116 B cell lymphoma-2 (BCL-2) AllKOBokKO cells transfected with individual EGFP-tagged BH3-only protein plasmids (the same cells as in Figure 1D) and integrated mean fluorescence intensity quantified using the in-built IncuCyte analysis software at 12h, 24h and 48h timepoints. All data were analysed using Anova comparing HRK to values from the other BCL-2 proteins transfected. Error bars represent standard error of 3 independent experiments. * = p<0.05, ** = p<0.01, **** = p<0.001.

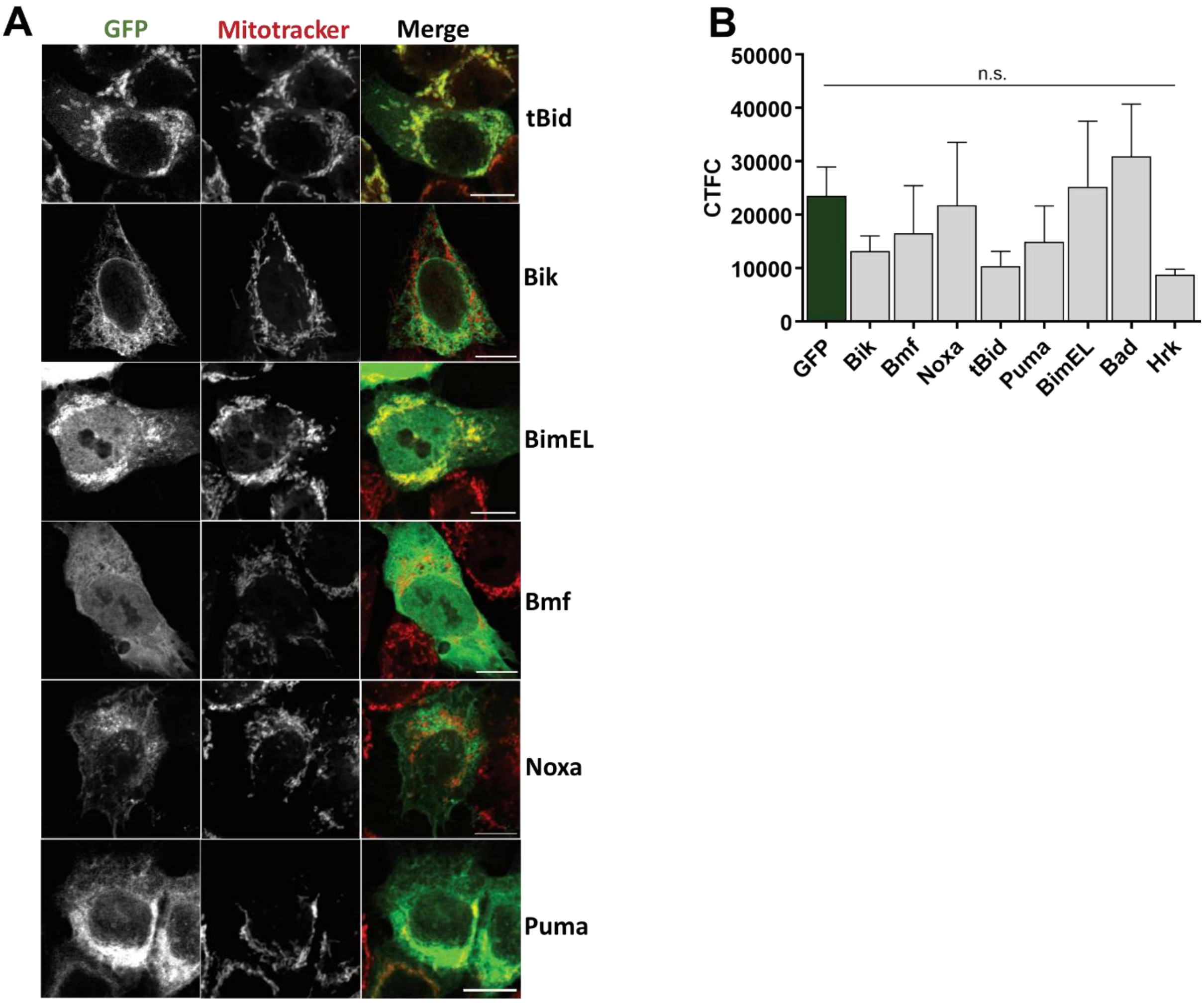

Figure S2 – Overexpression of BH3-only proteins other than HRK and BAD do not alter mitochondrial morphology. A. HCT116 B cell lymphoma-2 (BCL-2) AllKOBokKO cells transfected with individual enhanced green fluorescent protein (EGFP)-tagged BCL-2 homology (BH)3-only protein plasmids and stained with Mitotracker. B. Quantification of corrected total cell fluorescence (CTCF) of cells transfected with EGFP-tagged BH3-only proteins. Data represent n=75-100 cells from three independent experiments. Scale bars represent 10µm. Images were analysed using Fiji Image J. All data were analysed using Anova comparing to EGFP. Error bars represent standard deviation.

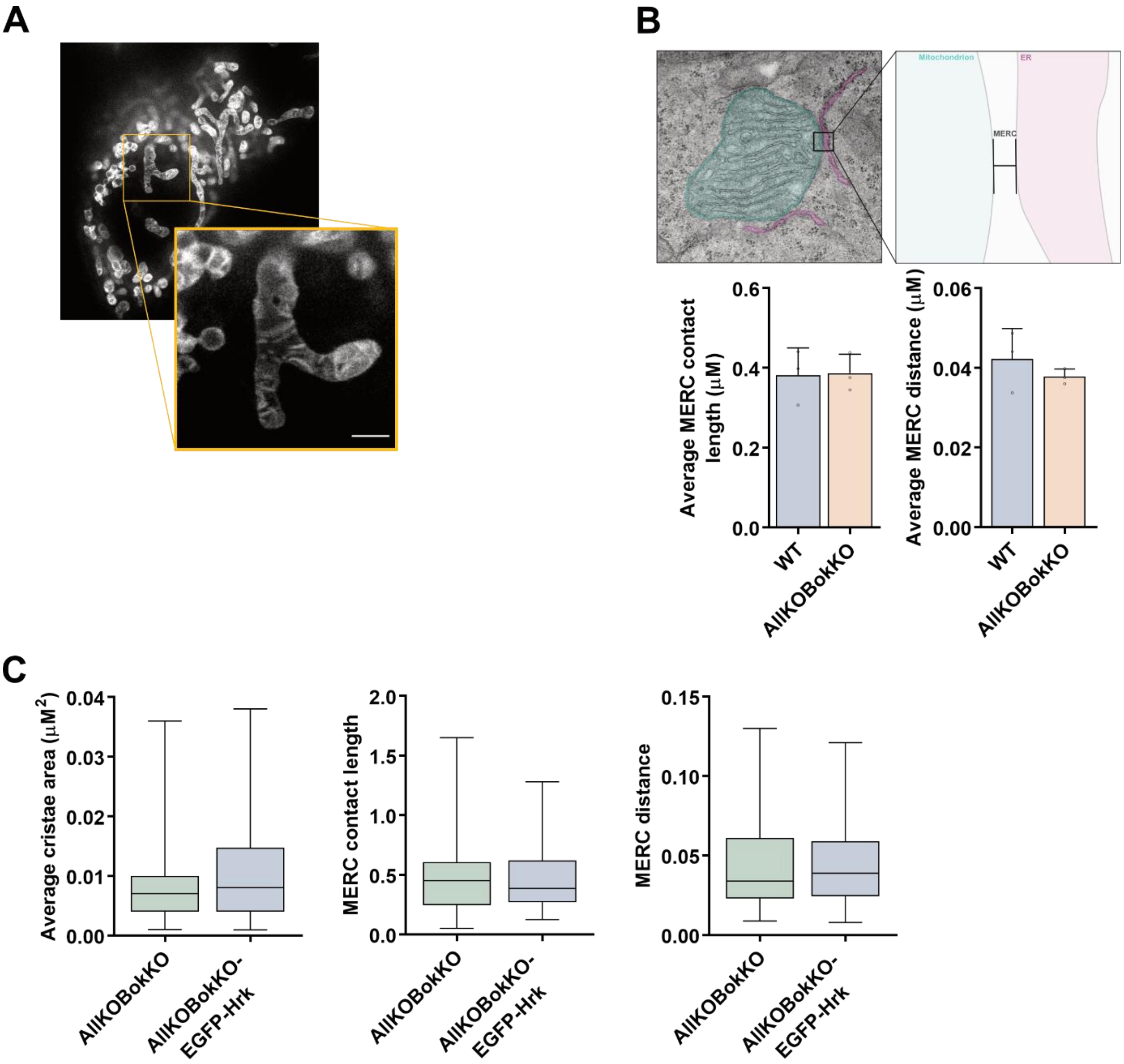

Figure S3 – HRK overexpression does not alter MERCs. A. Representative stimulated emission depletion image of HCT116 live cells stained with the mitochondrial inner membrane dye Pico Orange to visualise cristae structure. B. Representative image of a mitochondria- endoplasmic reticulum contact sites (MERC) from a transmission electron tomography image with corresponding analysis of contact length (end-to-end length across the MERC) and contact distance (length between ER and mitochondrion) in wildtype (WT) and HCT116 AllKOBokKO cells below. Data represents n= 80-100 mitochondria from three independent experiments. C. Analysis as in B comparing B cell lymphoma-2 (BCL-2) AllKOBokKO cells with AllKOBokKO-enhanced green fluorescent protein (EGFP)-HRK cells. Data represent values from 30 mitochondria from one biological experiment. All data were analysed using Anova.

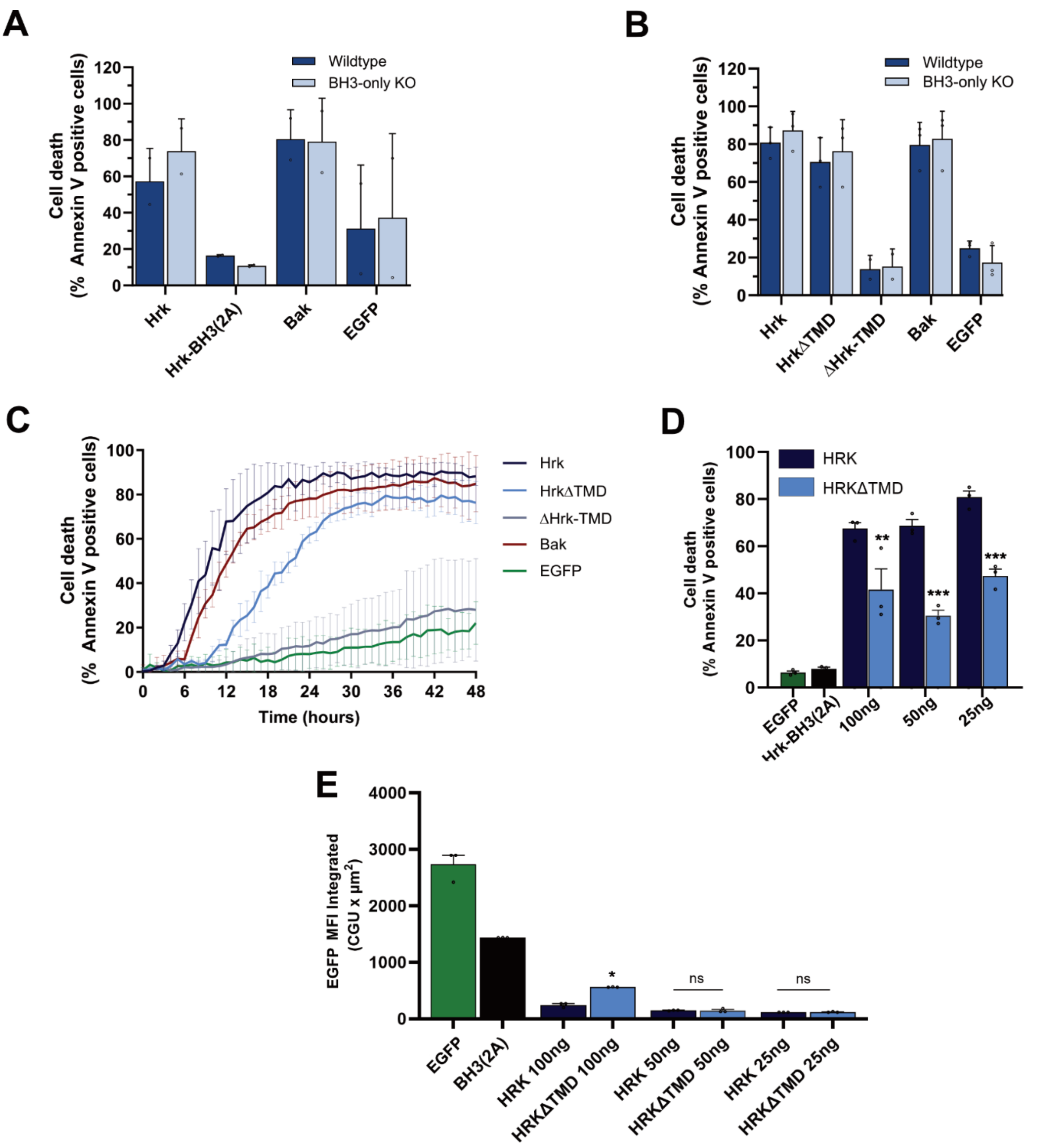

Figure S4 – BH3-only proteins do not affect HRK-induced cell death. A. 48-hour time point of IncuCyte cell death assays of wildtype (WT) and BH3-only knockout (KO) HCT116 cells transfected with either WT HRK, HRK-BH3(2A), BAK or EGFP, and cell death analysed over 48 hours. B. IncuCyte analysis as in A of cells transfected with either EGFP-tagged WT HRK, HRKΔTMD (HRK lacking its transmembrane domain), ΔHRK-TMD (only the TMD of HRK), BCL-2 antagonist/killer 1 (BAK) or enhanced green fluorescent protein (EGFP) and cell death analysed over 48 hours. Data were analysed

via Anova comparing BH3-KO values to WT. Error bars represent standard deviation of 3 independent experiments. C. Time course data from B showing rates of cell death. D. 48-hour time point of IncuCyte cell death assays of WT HCT116 cells transfected with 100ng of HRK-BH3(2A) and EGFP, and titrations of WT HRK and HRKΔTMD. Cell death analysed over 48 hours. E. The same cells as in D quantified for integrated mean fluorescence intensity using the in-built IncuCyte analysis software at the 48h timepoint. Data were analysed via Anova comparing full-length HRK to the TMD mutants. Error bars represent standard of 3 independent experiments. * = $p<0.05$, ** = $p<0.01$, *** = $p<0.005$.
